# Adaptation and plasticity in aboveground allometry variation of four pine species along environmental gradients

**DOI:** 10.1002/ece3.2153

**Published:** 2016-09-30

**Authors:** Natalia Vizcaíno‐Palomar, Inés Ibáñez, Santiago C. González‐Martínez, Miguel A. Zavala, Ricardo Alía

**Affiliations:** ^1^ Department of Forest Ecology and Genetics Forest Research Centre (INIA) Ctra. A Coruña, km 7.5 28040 Madrid Spain; ^2^ Forest Ecology and Restoration Group Department of Life Sciences Universidad de Alcalá Science Building Campus Universitario, 28871 Alcalá de Henares Madrid Spain; ^3^ School of Natural Resources and Environment University of Michigan Ann Arbor Michigan 48109; ^4^ Sustainable Forest Management Research Institute University of Valladolid‐INIA Avd. Madrid s/n 34004 Palencia Spain; ^5^ BIOGECO, INRA University of Bordeaux 33610 Cestas France

**Keywords:** Bayesian modeling, climatic and geographical clines, environmental gradients, functional trait, Iberian Peninsula, intraspecies variability, provenance tests

## Abstract

Plant species aboveground allometry can be viewed as a functional trait that reflects the evolutionary trade‐off between above‐ and belowground resources. In forest trees, allometry is related to productivity and resilience in different environments, and it is tightly connected with a compromise between efficiency‐safety and competitive ability. A better understanding on how this trait varies within and across species is critical to determine the potential of a species/population to perform along environmental gradients. We followed a hierarchical framework to assess tree height‐diameter allometry variation within and across four common European *Pinus* species. Tree height‐diameter allometry variation was a function of solely genetic components –approximated by either population effects or clinal geographic responses of the population's site of origin– and differential genetic plastic responses –approximated by the interaction between populations and two climatic variables of the growing sites (temperature and precipitation)–. Our results suggest that, at the species level, climate of the growing sites set the tree height‐diameter allometry of xeric and mesic species (*Pinus halepensis, P. pinaster and P. nigra*) apart from the boreal species (*P. sylvestris*), suggesting a weak signal of their phylogenies in the tree height‐diameter allometry variation. Moreover, accounting for interpopulation variability within species for the four pine species aided to: (1) detect genetic differences among populations in allometry variation, which in *P. nigra* and *P. pinaster* were linked to gene pools –genetic diversity measurements–; (2) reveal the presence of differential genetic variation in plastic responses along two climatic gradients in tree allometry variation. In *P. sylvestris* and *P. nigra*, genetic variation was the result of adaptive patterns to climate, while in *P. pinaster* and *P. halepensis*, this signal was either weaker or absent, respectively; and (3) detect local adaptation in the exponent of the tree height‐diameter allometry relationship in two of the four species (*P. sylvestris* and *P. nigra*), as it was a function of populations' latitude and altitude variables. Our findings suggest that the four species have been subjected to different historical and climatic constraints that might have driven their aboveground allometry and promoted different life strategies.

## Introduction

Aboveground allometry is considered a functional trait that links the changes in total height to those in stem diameter and reflects the evolutionary outcome in plant species dynamics for above and belowground resources (Hallé et al. 1978; King 1996). Both height and stem diameter are tightly associated with species foraging and resource allocation strategy (Tilman 1988; Poorter et al. 2012): while tree height reflects a strategy for securing carbon profit via light capture (Moles et al. 2009), stem diameter is closely related to mechanical support and water‐absorbing capacity (McMahon 1973; Niklas 1993; Bullock 2000). A finite set of allometric outcomes is then expectable, due to trade‐offs in plant allocation strategies along resource gradients (sensu Tilman 1988) or biomechanical and hydraulic constraints (e.g., Ryan and Yoder 1997; Chave et al. 2005; Mäkelä and Valentine 2006).

Tree height‐diameter allometry has profound effects on species fitness and consequently on ecosystem structure. It correlates with bioclimatic variables (e.g., Aiba and Kohyama 1996; López‐Serrano et al. 2005; King et al. 2006), and can change along biotic and abiotic gradients such as those for temperature, aridity, and competition (e.g., Banin et al. 2012; Lines et al. 2012). However, intraspecific variation in allometry has usually been neglected and most studies have focused either on the species level or on the broad geographical scales (Chave et al. 2005; López‐Serrano et al. 2005; Dietze et al. 2008; Lines et al. 2012; Poorter et al. 2012; but see Vieilledent et al. 2010; Pretzsch and Dieler 2012; that considered individual variability). The extent and patterns of variation in interpopulation genetic in tree height‐diameter allometry still remain unclear. Those patterns could be as a result of adaptive or neutral genetic processes, such as past events, for example, migration, bottlenecks, and drift, at different scales (species, population or individual), of plastic responses to the environment, or by any combinations of them. Consequently, aboveground allometry emerges as a comprehensive and integrative trait in which the pattern of allocation variation within species could be driven by climate and interpopulation genetic variation. A deep understanding of these interconnected levels of variability (species and populations) in tree height‐diameter allometry is necessary to forecast the full potential of tree species to adapt and/or evolve under climate change conditions (e.g., Benito‐Garzón et al. 2011; Valladares et al. 2014).

Common garden experiments are established for testing genetic differences among populations grown under similar environmental conditions and generate valuable information for the study of intraspecific genetic variation (e.g., Matyas 1996; Alberto et al. 2013). Multilocality common gardens, additionally, allow studying phenotypic plastic responses along environmental gradients and to identify genetic variation on them (i.e., population‐environment interaction) and the adaptive value of those responses as well (i.e., correlations between the growing environments and local environments of population's origin).

In this study, we used total height and stem diameter – over bark– measured in multilocality common garden tests to assess allometry relationships for the four most planted European pine species: *Pinus sylvestris* L., *P. nigra* Arnold*, P. pinaster* Aiton, and *P. halepensis* Miller. The first two species (*P. sylvestris* and *P. nigra)* belong to the *Pinus* subsection that corresponds to Eurasian pines; and the last two species to the *Pinaster* subsection which relates to Mediterranean pines. Accordingly, these species display differentiated demographic backgrounds and genetic compositions resulting in a predictable pattern along temperature and water availability gradients across Europe (Richardson 1998; Tapias et al. 2004; Soto et al. 2010). We implemented a flexible log‐linear model taking into consideration each species' population origin and associated geographic characteristics (to account for intraspecific genetic variation), and the climatic characteristics of the growing site (to account for the among‐site variation). Correspondingly, we tested three hypotheses: (1) the patterns of height‐diameter allometry variation in pines are driven by both the species and the interpopulation variation; (2) at the species level, tree allometry will vary depending on the climatic characteristics of the species, and especially with marked differences among Eurasian an Mediterranean species; (3) interpopulation variation in allometry could be the result of adaptation to local environments –namely climate and/or geographical variables of populations' site of origin– or historical events that took place in the past of the species. Testing these hypotheses will allow us to understand the underlying abiotic drivers that shape allometry variation at two interconnected levels, species and populations, and to identify the class of adaptive responses if existent. Understanding phenotypic integration of tree species responses along abiotic conditions could then assist in forecasting the performance of forest species and populations in the context of global warming.

## Methods

### Plant material and common garden provenance tests

**Figure 1:**
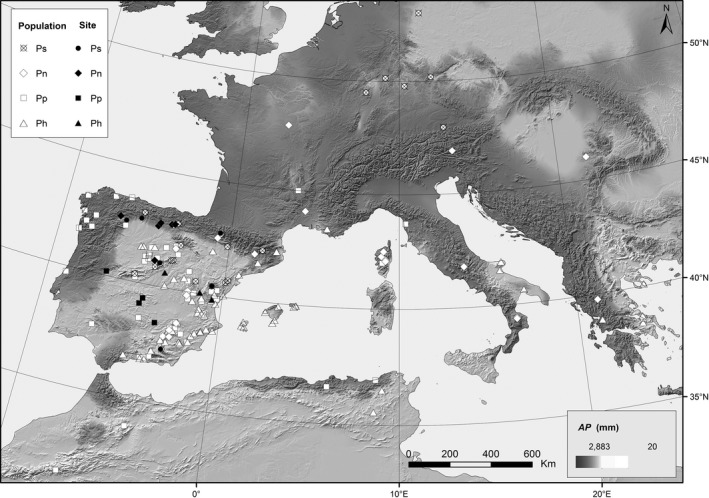
Common garden sites (Site, filled symbols), and population's sites of origin (Population, unfilled symbols) are represented in the map. Each pine species is represented in a different symbol Ps: *Pinus sylvestris*, and it is represented by a circle; Pn: *Pinus nigra,* it is represented by a rhomb; Pp: *Pinus pinaster* by a square, and Ph: *Pinus halepensis* by a triangle.

Aboveground allometry was measured in multilocality common garden provenance tests located in Spain for four pines species: *Pinus sylvestris*,* P. nigra*,* P. pinaster,* and *P. halepensis*. Populations from the distribution range of the species, mostly from the Iberian Peninsula (Spain and Portugal), were sampled by collecting seed lots from at least 25 mother trees with a 50‐m separation distance. Plants originating from the seed lots were collected in different populations (22 for *P. sylvestris*, 23 for *P. nigra*, 52 for *P. pinaster,* and 56 for *P. halepensis*) and established in comparative common garden provenance tests for each species (Fig. 1 and see Table S1 in Supporting Information).

Measurements were collected at 11 ± 1 years of age, depending on the common garden tests, for two variables: *height* (total height in cm, measured with a pole) and *dbh* (diameter at breast height [130 cm] in mm, measured with a caliper). A common age was chosen to minimize species differences along their ontogenies (López‐Serrano et al. 2005), and avoid confusion of allometric changes due to size, known as ‘passive or apparent plasticity’ (Wright and Mcconnaughay 2002). We also selected a young age to minimize interpopulation competition effects in the experimental design. A previous study using the same experiment setup as in the present research did not find either inter‐ or intrapopulation competition effects in any of the two variables measured (*height* and *dbh*) in 32‐year‐old *P. pinaster* individuals (Alía et al. 2001).

In total, we used data from 4853 *P. sylvestris* trees from 22 populations planted in 6 sites; 3644 *P. nigra* trees from 23 populations in 8 sites; 9976 *P. pinaster* trees from 54 populations in 4 sites; and 1928 *P. halepensis* trees from 56 populations in 3 sites.

### Climatic and geographical data

Each site was characterized by a set of 47 climatic variables: minimum, average and maximum mean monthly temperature, minimum and maximum average temperature of the coldest and warmest months, and total and seasonal precipitation. As we lacked real climate data from weather stations, we estimated these variables based upon Gonzalo‐Jiménez's (2010) climatic model for the Iberian Peninsula, with a 1‐km^2^ spatial resolution, from climate data gathered between 1951 and 1999 (see Appendix S1 for further information).

According to both literature and exploratory analyses, we selected the subset of climatic variables at the growing sites most relevant to plant allometry for the four species. The selected variables were MMT (minimum average temperature of coldest month, °C) and AP (annual precipitation, mm). Both MMT and AP affect physiological and growth processes of plant species in the Mediterranean region (Thompson 2005) and have been consistently used in previous studies (e.g., Wang et al. 2006; O'Neill and Nigh 2011; Leites et al. 2012). Moreover, these variables presented substantial correlation with *height* and *dbh* variables (see Table S3). Geographical variables of the populations' site of origin, such as latitude, longitude and altitude, are surrogates for environmental conditions, for example, the amount of heat energy received relative to the sun angle, temperature, humidity, and solar radiation; and they can usually reflect adaptation patterns to local conditions (see Alberto et al. 2013). Climatic variables of the growing sites and geographic variables of the populations' site of origin were then standardized before analyses to ease comparison among variables in the model.

Although the number of growing sites is low (ranging between 3 and 8), they cover most of the natural climatic range associated with each species distributional range, including contrasting climates (Ruiz‐Benito et al. 2013) (see Fig. S1).

### Statistical models

We estimated tree height as a function of diameter using three classic allometric functions (Linear, Power, and Gompertz), and two link functions (normal and lognormal) and implemented generalized linear models (GLMs). The best allometric model fitting the data was selected using the Deviance Information Criteria, DIC (Spiegelhalter et al. 2002). A power function with a lognormal link function was the best model for two of the four species, and the second best model for the other two species (see Table S2). We selected a common allometric model, power function with a lognormal link, for the four species to facilitate parameter comparisons.

Based on this allometric model, we constructed a hierarchical model (Clark 2005, 2007). These models are more appropriate to connect and represent the biological hierarchy of the data, for example, populations within species. To build the best final model, we considered several variations of the basic model (i.e., in equation 1), where *a* and *c* scaling parameters were constant, and they were estimated with different combinations of the variables associated with the growing sites and the origin of populations. The best final model structure was selected based on both biological relevance and the DIC criterion.

The final model estimated tree height allometry as a combination of climate at the growing site (*s*) and geographic characteristics at the origin site of the population (*p*).

Considering an individual *i*, from population *p* growing in growing site *s*, its height‐diameter allometry was modeled as:

Likelihood: height_*i*_ ~ log Normal (*H*_*i*_ *, σ* ^*2*^) and the following process model:

(1) $$H_{i}=\;\text{ln}\;\left(a_{p_{(i),}s_{\left(i\right)}}\right)+c_{p_{\left(i\right)}}\times dbh_{i}$$

where the scaling coefficient $\;\text{ln}\;(a_{p_{(i),}s_{\left(i\right)}})$ was estimated as:

(2) $$\;\text{ln}\;\left(a_{p_{(i),}s_{\left(i\right)}}\right)=\alpha_{1p}+\alpha_{2p}\times \;{\text{MMT}}_{s}+\alpha_{3p}\times {\text{AP}}_{s}$$

and the scaling exponent, $c_{p_{\left(i\right)}}$, was estimated as:

(3) $$c_{p_{\left(i\right)}}=\beta_{1}+\beta_{2}\times \;{\text{LAT}}_{p}+\beta_{3}\times {\text{ALT}}_{p}$$

**Figure 2:**
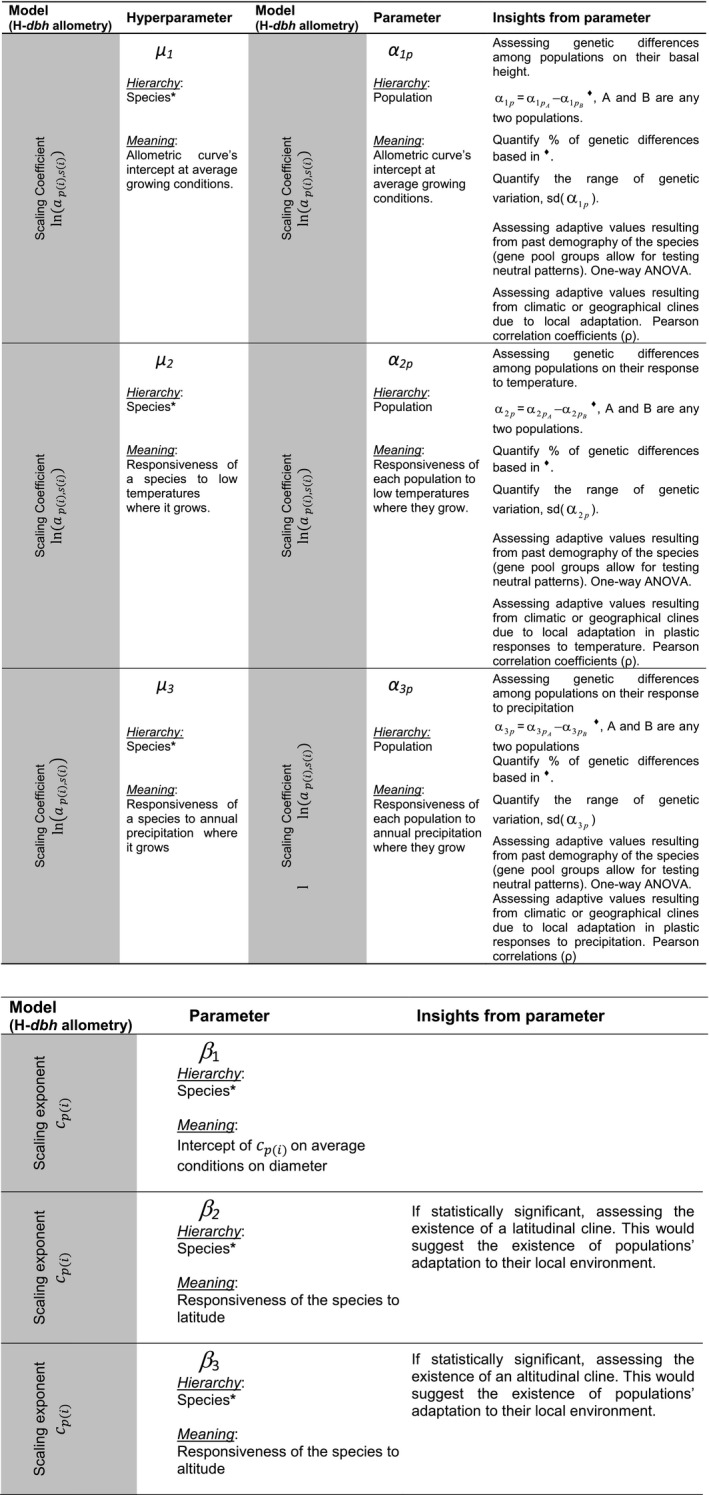
Summary information of the estimated parameters in the final tree height‐diameter allometry model. We have described each parameter attending to its hierarchy, its significance and the set of research questions that can be addressed. *In this study, the species term is approximated as the average response calculated taking into account the set of populations considered in the current study.

Tree height‐diameter allometry, therefore, is the outcome result of population genetic effects on the basal height, parameterized in *α*_*1p*_; plus a genetic (population) clinal geographical pattern of the scaling exponent on latitude and altitude (*β*_2_ × *LAT*_*p*,_ *β*_3_ × *ALT*_*p*_), and of genetic differential plastic responses along temperature and or precipitation gradients of the growing site (*α*_*2p*_ *× MMT*_*s*_,* α*_*3p*_ *× AP*_*s*_). Because all explanatory variables were standardized, parameter *α*_*1p*_ was the allometric curve's intercept at average climate conditions of across all growing sites; and likewise holds for *β*_1_. A summary of model parameters, significance, and insights that can be assessed on each one is shown in Figure 2.

### Model parameters estimation and post hoc comparisons

Parameters were estimated following a Bayesian approach highly suited for hierarchical analyses (Gelman and Hill 2007). Each of the population level parameters, $\alpha_{*p}$, was estimated from a species‐level prior normal distribution, with hyperparameters μ_*_ and $\sigma_{\alpha *}^{2},\alpha_{*p}∼N\left(\mu *,\sigma_{\alpha *}^{2}\right)$, estimated from noninformative prior distributions μ_*_ ~ *N* (0, 1000) and *σ*_α*_ ~ Uniform (0, 100). These species‐level parameters μ_*_ and $\sigma_{\alpha *}^{2}$ would correspond sensu stricto to an interpopulation average among the studied populations. However, we refer to these parameters sensu lato as representative of a species proxy response. Note that for the scaling coefficient we have referred in equation 2 to populations' parameters to enhance comprehension of the full scope of the relationship, instead of including the species' parameters.

Parameters β_*_ were also estimated from noninformative prior distributions, β_*_ ~ *N* (0, 1000). Variance associated with the individual random effects was estimated as 1/*σ* ^2^ ~ Gamma (0.01, 0.01). As standard deviation of residual errors around a fitted power function might increase with diameter, we tested whether the residuals were a linear function of diameter, as recommended in Lines et al. (2012). However, our residuals did not show this trend, so we considered unnecessary to account for diameter size in the estimation of the variance.

We formally tested marginal significant intraspecific genetic differences in $\alpha_{*p}$ for each species by computing all pairwise combinations of population differences accounting for the 95% credible interval, CI, of the estimated parameter distribution (e.g., intraspecific genetic differences in $\alpha_{*p}=\alpha_{*pA}-\alpha_{*p_{B}}$, being *A* and *B* two populations of a specific pine species), while the rest of variables were kept to their mean values in the range, that is why we refer to these differences as “marginal”. Two populations were significantly different if zero was not included in the credible interval around their difference. Additionally, we quantified the level of marginal intraspecific genetic differences as the percentage of the total number of significant pairwise comparisons relative to the total number of pairwise comparisons within species. Finally, to end the characterization of intraspecific genetic variability within species, we provided the range of variability among populations within species as the standard deviation of $\alpha_{*p}$, that is, the set of parameters estimated for each population.

Models were run in OpenBUGs (version 3.2.2 rev 1063) (Thomas et al. 2006). Three chains were run for ~50,000 iterations, and parameters convergence was reached after ~25,000 iterations. After the burn‐in period, chains were thinned (every 100) to reduce autocorrelation, and then, posterior parameter values (mean and 95% credible intervals) were calculated. Plots of predicted vs. observed values were also used to evaluate model fit (unbiased models having a slope of one and *R* ^*2*^ values indicating goodness of fit). A slope parameter was considered to be statistically significant when the 95% credible interval (CI) did not include zero. Population level parameters were considered significantly different when their 95% CI did not overlap (or the 95% CI around their difference did not include zero).

### Species' adaptive patterns in height–diameter allometry variation

We tested whether variation in the $\alpha_{*p}$ parameters was the result of neutral and/or adaptive responses to local environments, using two different approaches. First, to assess the influence of neutral responses on $\alpha_{*p}$ parameters of allometry, we tested the influence of previously defined gene pools groups for each species on $\alpha_{*p}$ parameters. Gene pools are genetic groups of populations based on neutral molecular markers. Populations within the same genetic group is likely to share the same evolutionary history in terms of origin of the same glacial refuges and colonization routes. In *P. sylvestris*,* P. pinaster* and *P. halepensis*, gene pool groups were defined using molecular markers in Robledo‐Arnuncio et al. (2005) and in Bucci et al. (2007). In *P. nigra*, we lacked information based on molecular markers; hence, populations were grouped by subspecies. One‐way ANOVA was used to detect the existence of association between $\alpha_{p}^{*}$ parameters and groups, and post hoc comparisons with a Tukey's HSD test were employed. When homogeneity and normality assumptions were not reached, nonparametric Kruskal–Wallis test and post hoc comparisons with a Nemenyi test, corrected for ties if necessary, were used. Second, to identify the influence of local environments – namely climate and/or geographical position of populations' site of origin– on tree allometry variation, we tested the existence of climatic or geographical clines due to local adaptations in phenotypic plasticity. More specifically, we computed Pearson's correlation coefficients (*ρ*) between $\alpha_{p}^{*}$ parameters and climate and geographical characteristics of the populations' site of origin.

## Results

### Patterns of height–diameter allometry variation across and within species

The four final models produced unbiased estimates of *height* with high *R* ^*2*^ of observed *vs* predicted values (0.90 in *P. sylvestris*, 0.91 in *P. nigra*, 0.85 in *P. pinaster,* and 0.89 in *P. halepensis*).

*Pinus pinaster* had the lowest intercept value, measured by the hyperparameter *μ*_1,_ and it did not overlap with the other three species. *P. nigra* and *P. sylvestris* had intermediate but overlapping values, while *P. halepensis* had the highest value and did not overlap with any of the other pine species (Table 1). The intraspecific genetic variability, the standard deviation of *α*_1*p*_, also varied among species. *P. pinaster* displayed the greatest value, followed by *P. nigra*,* P. sylvestris,* and *P. halepensis* (Table 1). This intraspecific genetic variability can also be visualized in Figure S2. Moreover, there was a statistical significant intraspecific genetic variation in *α*_1*p*_ in all species, marginal significant intraspecific genetic differences measured by the number of significant pairwise comparisons within species relative to the total number: *P. pinaster* was the species with the highest percentage of significant differences (50.24%); followed by *P. nigra* (40.32%), *P. sylvestris* (31.17%), and *P. halepensis* (17.21%).

**Table 1:** Parameter estimates from the selected best model. The table gathers information of two interconnected levels of hierarchy, species, and populations

		*P. sylvestris*	*P. nigra*	*P. pinaster*	*P. halepensis*
*ln*(*a*_*p,s*_)	Intercept: *μ*_1_	**4.143** b **[4.123, 4.126]**	**4.121** b **[4.093, 4.148]**	**3.056** c **[3.023, 3.088]**	**4.344** a **[4.302, 4.385]**
	*α*_*1p*_	[4.107 … 4.229]	[4.047 … 4.198]	[2.907 … 3.170]	[4.281 … 4.396]
	sd(*α*_*1p*_)	0.028	0.039	0.054	0.024
	*MMT*:* μ*_2_	**−0.030** d **[−0.049, −0.011]**	**0.037** c **[0.026, 0.049]**	**0.086** b **[0.072, 0.100]**	**0.158** a **[0.143, 0.172]**
	*α*_*2p*_	[**−**0.120 … 0.026]	[0.014 … 0.063]	[**−**0.002 … 0.169]	[0.143 … 0.174]
	sd(*α*_*2p*_)	0.032	0.011	0.026	0.007
	*AP*:* μ*_3_	**0.080** a **[0.070, 0.095]**	**−0.0184** b **[−0.028, −0.009 ]**	**−0.023** bc **[−0.036, −0.009]**	**−0.049** c **[−0.064, −0.035]**
	*α*_*3p*_	[0.043 … 0.128]	[**−**0.032 … 0.000]	[**−**0.059 … 0.032]	[**−**0.053 … **−**0.043]
	sd(*α*_*3p*_)	0.019	0.007	0.018	0.002
*c*_*p*_	Intercept: *β*_0_	**0.426** b **[0.416, 0.431]**	**0.412** bc **[0.405, 0419]**	**0.700** a **[0.691, 0.707]**	**0.397** c **[0.375, 0.409]**
	*LAT*:* β*_1_	**0.016** a **[0.007, 0.026]**	**0.010** ab **[0.002, 0.017]**	0.005 [**−**0.001, 0.012]	0.003 [**−**0.003, 0.008]
	*ALT*:* β*_2_	**−**0.006 [**−**0.015, 0.004]	**−**0.004 [**−**0.011, 0.004]	**−**0.007 [**−**0.013, 0.000]	**−**0.002 [**−**0.007, 0.004]

The parameters μ_*_ and β_*_ make reference to the species, i.e., the species‐level, and α_*p_ to populations within species, i.e., population‐level. The overall species response, that is, μ_*_ and β_*_ posterior mean estimates and 95% credible intervals in square brackets [, ] are given. The range of parameter values among populations within species, that is, posterior mean estimates of α_*p_, are shown in square brackets, the lowest value is separated from the highest one by three dots [ … ]. Bold numbers indicate that fixed‐effect coefficients were statistically significant (i.e., 95% CI does not include zero). Letters indicate comparison and different letters indicate differences among species for each of the parameters when statistically significant.

Temperature (MMT) of the growing site influenced tree height allometry, being this hyperparameter, *μ*_2_, statistically significant and positive in three of the four species, and significant but negative in *P. sylvestris* (Table 1). Moreover, we found evidence of intraspecific genetic differences in phenotypic plasticity to temperature (MMT) in three of the four species (except *P. halepensis*). Accordingly, the four species showed some degree of intraspecific genetic variability, *P. sylvestris* having the greatest standard deviation, followed by *P. pinaster*,* P. nigra,* and *P. halepensis*. More specifically, the level of significant intraspecific genetic variation varied according to each species. Thus, *P. sylvestris* displayed the greatest level of genetic differences in plasticity in response to MMT (38.10%) among the populations tested, followed by the other two species: *P. nigra* (9.88%) and *P. pinaster* (9.57%). This intraspecific genetic variability can also be visualized in Figure S3. All these results should be considered based on the total of populations tested.

Annual precipitation (AP) also influenced tree height allometry. Values for hyperparameter *μ*_3_ were statistically significant and negative in three of the four species, but positive in *P. sylvestris*. The estimated values for *P. sylvestris* and *P. halepensis* did not overlap, but the pairs composed by *P. nigra* and *P. pinaster*, and *P. pinaster* and *P. halepensis* did (Table 1). Similarly, we found intraspecific genetic differences in phenotypic plasticity to rainfall (AP) in three of four species, the exception again being *P. halepensis*. Furthermore, the four pine species presented some degree of intraspecific genetic variability. *P. sylvestris* and *P. pinaster* presented similar degrees, followed by *P. nigra* and *P. halepensis* (Table 1). The level of significant intraspecific genetic variation was greatest in *P. sylvestris* (29.87%), followed by *P. pinaster* (3.60%) and *P. nigra* (2.77%). This intraspecific variability can also be visualized in Figure S4.

In three of the four species, the effects of AP on the tree height‐diameter relationship and also the intraspecific genetic variability were smaller than those reported in response to MMT; specifically, between *ca*. 2 and 3.5‐folds greater for *P. nigra, P. halepensis,* and *P. pinaster* – in an increasing order–. Interestingly, the opposite effect was found in *P. sylvestris* –the effect of AP was ca. 2.5‐folds greater than MMT.

### Species' adaptive patterns in height–diameter allometry variation

Overall, we found that tree height allometry variation was the result of adaptive responses to either local environments –climate and geographical sites of origin –or to past historical events in the demography of species. First, we found a significant geographical cline, that is, an association between the scaling exponent parameter (*c*_*p*_) and the latitude of origin for two of the four pine species (*P. sylvestris* and *P. nigra*), but not for the other two, more xeric, species (*P. pinaster* and *P. halepensis*) (Table 1). Second, gene pool groups were significantly associated with *α*_*1p*_ values just in *P. pinaster* and *P. nigra* (*P* < 0.001 and *P* < 0.05, respectively), but not in the two others (Table 2).

**Table 2:** (A) Summary of one‐way ANOVAs to test gene pool effects on α_*p_. When a nonparametric test was used, it is shown by the symbol^≈^. (B) Post hoc comparisons among gene pools adjusted by Tukey's HSD for *Pinus nigra* and *Pinus pinaster*. Different letters indicate differences among gene pools

(A)			
Species	Parameter	*F*/*K*	*P*‐value
*Pinus sylvestris*	*α*_*1p*_	0.60	n.s.
	*α*_*2p*_	0.57	n.s.
	*α*_*3p*_	0.57	n.s.
*Pinus nigra*	*α*_*1p*_	6.95	**
	*α*_*2p*_	7.20^≈^	n.s.
	*α*_*3p*_	2.53^≈^	n.s.
*Pinus pinaster*	*α*_*1p*_	12.43	***
	*α*_*2p*_	14.23^≈^	n.s.
	*α*_*3p*_	3.84^≈^	n.s.
*Pinus halepensis*	*α*_*1p*_	1.07	n.s.
	*α*_*2p*_	0.44	n.s.
	*α*_*3p*_	0.08	n.s.

(B)			
Species	Gene pools	*α*_*1p*_	SD
*Pinus nigra*	spp. *laricio*	4.15	0.04 a
	spp. *salzmannii*	4.12	0.01 ab
	spp. *dalmatica*	4.08	a ab
	spp*. nigra*	4.06	0.02 b
*Pinus pinaster*	Morocco	3.11	0.00 a
	Atlantic Iberian	3.10	0.02 a
	Eastern Spain	3.06	0.03 ab
	Southern Spain	3.06	0.05 abc
	Corsica	3.05	0.00 abc
	Central Spain	3.02	0.03 bc
	Italy	2.95	a cd
	Eastern North Africa	2.91	0.00 d

a Only one datum, standard deviation was not estimated.

Third, we found chiefly greater association between the local environment and parameter *α*_*1p*_ compared to *α*_*2p*_ and *α*_*3p*_. Here the local environment is understood as the set of variables that define the climate of the sites of origin of populations, through proper climate variables or based upon the geographical origin of them. Interestingly, *P. halepensis* was the only species that lacked any type of relationship, suggesting the inexistence of climate adaptive responses in tree allometry variation. Specifically, *α*_*1p*_ values were significantly correlated (*P *<* *0.05) to different climatic variables of population's site of origin (Table 3). *P. sylvestris* with altitude (*ρ *= 0.56) and annual precipitation (*ρ *= 0.54); *P. nigra*, in general, with minimum average monthly temperature (ranging from *ρ *= 0.45 to 0.60); and, weaker than the previous two, *P. pinaster* with spring precipitation (*ρ *= 0.28) and mean temperature of the warmest month (*ρ *= –0.28). Parameters *α*_*2p*_ and *α*_*3p*_ were significantly correlated with climatic variables of populations' sites of origin (*P *<* *0.05); *P. sylvestris*,* P. nigra,* and *P. pinaster* displayed significant correlations between *α*_*2p*_ and climate, although the associations were weaker in the last species. Finally, we only found significant and positive correlations between *α*_*3p*_ and related temperature variables in *P. sylvestris* (Table 3).

**Table 3:**
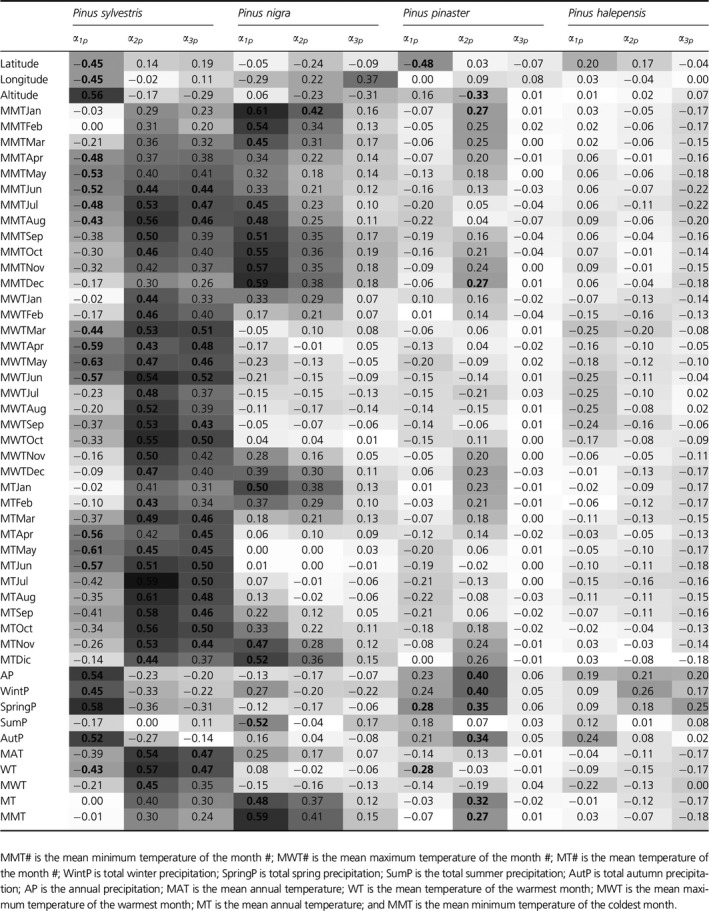
Heat map for Pearson's correlation coefficients, *ρ*, between α_*p_ and climate variables from the populations' sites of origin. Dark gray indicates high positive correlation coefficients, light gray indicates high negative, and white color indicates low. Bold numbers mean significant correlations at *P* < 0.05

## Discussion

We addressed interpopulation tree height‐diameter allometry variation across precipitation and temperature gradients of the four most planted pine species in Spain; that is, tree height‐diameter allometry variation was accounted at two interconnected levels, species and population. Additionally, we found that local adaptation and past historical events of the species were associated to interpopulation allometry variation, except for *P. halepensis,* the most xeric species among the four. We employed a hierarchical approach to better understand the contributions of the species' genetic variation, their demographic genetic background and their phenotypic plasticity, in their responses to environmental variability.

### Patterns of height–diameter allometry variation across and within species

This is the first time, to our knowledge, that the patterns of aboveground allometry across climatic gradients have been described including intraspecific variation from a genetic perspective. The species‐level parameters related to allometry (hyperparameters μ_*_ and β_*_ *,* Fig. 2) did not overlap among species in most comparisons, reflecting the existence of evolutionary species‐specific allocation strategies to cope with the current environment, although revealing an unclear association with their phylogeny, because *P. nigra* behaved more like *P. halepensis* and *P. pinaster* than like *P. sylvestris*.

In general, *P. sylvestris* showed the opposite pattern in regard to aboveground allometry variation across precipitation and temperature gradients with respect to the other three species. Aboveground variation was also more responsive to changes in the minimum average temperature of the coldest month, MMT, than to changes in annual precipitation, AP (excluding again *P. sylvestris*). This is contrary to expectation as Iberian forests are strongly constrained by water availability (Gómez‐Aparicio et al. 2011; Ruiz‐Benito et al. 2013). We hypothesize that mesic and xeric pine species could be more limited by low temperatures than by water shortage, as they may have developed adaptive mechanisms to cope with drought stress, such as tight stomatal control, or specific wood anatomy traits such as thick cell walls, thick pit membrane, narrow lumens, or different root hydraulic resistance (Yastrebov 1996; Tyree and Zimmermann 2002; García Esteban et al. 2009; Zuccarini et al. 2015). Yet our data did not allow us to explore all potential interactions, for example, too cool‐too wet; too warm‐too wet that are likely to shape evolutionary responses in these species and populations.

Consistently with previous studies, in three of the four pine species, taller heights at a given size are found under warmer conditions (Lines et al. 2012), except for *P. sylvestris*. Warmer conditions during the cold season might allow a higher photosynthetic capacity, resulting in a higher rate of carbon assimilation (Way and Oren 2010), and hence taller heights. Yet *P. sylvestris* showed the opposite trend, suggesting a lack of responsiveness to warmer winters. A similar result was reported by Reich and Oleksyn (2008) in a regional study in Northern Europe (latitude from 46° to 68° N). They observed that *P. sylvestris* responses to climate differed between northern and southern populations: while in southern populations height decreased as temperature increased, the opposite was observed in northern populations. It was suggested that, at least for this temperate‐boreal species, warmer temperatures –at its warmer range– might rather enhance heat stress and heat‐induced moisture stress than alleviate cold stress.

Tree height allometry variation across the precipitation gradient resulted species‐specific and diverse. *P. sylvestris* is expected to decrease its height at given size under drier conditions, a common pattern found in many parts of the world, for example, Méndez‐Alonzo et al. (2008). This variation has often been attributed to the changing hydraulic structure of vessels in drought‐prone areas. The opposite, however, was observed for *P. halepensis*. Periods of soil moisture saturation and flooding may act as stressors in arid‐climate forests by reducing tree height (Rodríguez‐González et al. 2010). Also higher precipitation levels in some regions could imply poorer soil quality, because of increased runoff and nutrient leaching. However, as we do not have these measurements, we cannot confirm its potential influence. Intermediate patterns in tree allometry variation were shown for *P. pinaster* and *P. nigra*, which displayed negligible variation along the precipitation gradient tested. Lines et al. (2012) found a clear pattern of allometric variation across species along the studied precipitation gradient, although not within species. That finding together with ours suggests that tree height allometry variation could have a very conservative performance across precipitation gradients. This latter would be in agreement with the results presented in Table 1. Here, the estimated credible intervals, CI, for the hyperparameters in *P. pinaster* and *P. nigra* were very close to containing zero. This is somehow reflecting the almost lack of influence of precipitation on tree height‐diameter allometry variation.

### Species' adaptive patterns in height–diameter allometry variation

According to our findings, interpopulation tree height allometry variation was the result of local adaptation (Table 3). In addition, for *Pinus nigra* and *P. pinaster*, the demographic history of the species associated with distinct neutral gene pools was also important (as they reflect past events along the species' history, such as genetic bottlenecks, founder effects, drift, etc.; Bucci et al. 2007; Soto et al. 2010; Jaramillo‐Correa et al. 2015) (Table 2). Thus, in these two species, gene pool groups correlate with its allometry –its phenotype, and therefore, gene pool groups could be further used for the study of different evolutionary processes on phenotype variation, although the delineated groups were appreciably different compared to those based on DNA data (Afzal‐Rafii and Dodd 2007; Bucci et al. 2007)–. Absence of this signal in *P. sylvestris* could be partly explained by a greater influence from local environments relative to species historical demographic background, as it is reflected by significant correlations between α_*p_ and local population of the species climate. In contrast, *P. pinaster* and *P. nigra* presented weak signals of adaptation to climate, specifically in plastic responses to temperature. Finally, *P. halepensis* represented a different case; its null degree of genetic variation, in any of the parameters of the model, agrees with the fact that the species' European Western populations of the species are genetically uniform (Soto et al. 2010), due to a relatively recent long‐range colonization from its ancestral range in the eastern Mediterranean Basin (Grivet et al. 2009).

The clear latitudinal variation in the scaling exponent parameter in *P. sylvestris* and *P. nigra* reveals a consistent regional correlation in tree allometry and photoperiod. Previous studies along latitudinal gradients have also found a genetic cline of adaptation (e.g., northern populations set buds and hardened earlier, and presented lower growth rates than the southern ones; see Alberto et al. 2013 and references therein). In any case, the lack of latitudinal clines in *P. halepensis* and *P. pinaster* could be explained to either insufficient span in our data or to a real lack of latitudinal variability. Interestingly, this is the first time that adaptive patterns have been shown for a composite trait such as tree height allometry. Our results confirm that this trait and its confined variation may be under natural selection control and consequentially play an important role in both the adaptation and acclimation potential of tree species to future conditions.

In conclusion, these four pine species are a heterogeneous group with a recognized ability to adapt to extremely variable environments. Our findings support the eco‐evolutionary knowledge we already have about them, but nonetheless reveal that tree height‐diameter allometry variation patterns have developed under different natural selection pressures, despite the study species sharing a sizeable part of their distribution area in the studied region. This might have resulted in species, such as *P. halepensis*, where phenotypic plasticity is more important than genetic variation; while for others, for example, *P. pinaster*, genetic variation and local adaptation might be more relevant. Together, local environments –at the origin– and current growing conditions outline the likely possible outcomes of integrated phenotypes.

The full potential of forest resilience and resistance along new temperature and aridity gradients, that is, climate change driven, would depend on local adaptation and levels of phenotypic plasticity of the populations. Our results point that considering both the species specific and population ecological and historical background is a key for assessing likely population responses to environmental variation.

## Data Accessibility

All the data used are available in the Database on Genetic trials GENFORED (www.genfored.es) upon request, and also, it is in process of submitting into the CitaREA repository (http://citarea.cita-aragon.es/citarea/?locale=en). Identificators for the data will be available before publication.

## Conflict of Interest

None declared.

## Supporting information


**Appendix S1.** Appropriateness of the use of estimated climate data instead of long‐term average data originated in weather stations.
**Table S1.** Characteristics of the common garden experiments, i.e., growing sites.
**Table S2.** Summary of the different allometric and linking functions tested for each species.
**Table S3.** Heat map for Pearson's correlation coefficients, *ρ*, between *height* and *dbh*, together with the climatic and geographic variables from the common garden sites for the four species.
**Figure S1**. Principal Component Analysis (PCA) of pine populations (P) and growing sites (S).
**Figure S2.** The figure shows α_*1p*_ values for the four pine species studied, and their 95% credible intervals.
**Figure S3.** The figure shows α_*2p*_ values for the four pine species studied, and their 95% credible intervals.
**Figure S4.** The figure shows α_*3p*_ values for the four pine species studied, and their 95% credible intervals.
