# Supplementary material for: Adaptation and plasticity in aboveground allometry variation of four pine species along environmental gradients
Source: Ecol Evol. 2016 Sep 30;6(21):7561–73. doi: 10.1002/ece3.2153 (PMC6512899; doi:10.1002/ece3.2153)
Supplement: Supplementary file 1 — Appendix S1. Appropriateness of the use of estimated climate data instead of long‐term average data originated in weather stations. Table S1. Characteristics of the common garden experiments, i.e., growing sites. Table S2. Summary of the different allometric and linking functions tested for each species. Table S3. Heat map for Pearson's correlation coefficients, ρ, between height and dbh, together with the climatic and geographic variables from the common garden sites for the four species. Figure S1. Principal Component Analysis (PCA) of pine populations (P) and growing sites (S). Figure S2. The figure shows α1p values for the four pine species studied, and their 95% credible intervals. Figure S3. The figure shows α2p values for the four pine species studied, and their 95% credible intervals. Figure S4. The figure shows α3p values for the four pine species studied, and their 95% credible intervals. [file ECE3-6-7561-s001.docx]

**Supplementary Materials to the paper**

Vizcaíno-Palomar, N. et al. Adaptation and plasticity in aboveground allometry variation of four pine species along environmental gradients. *Ecology and Evolution*.

**Appendix text S1.** Appropriateness of the use of estimated climate data instead of long-term average data originated in weather stations.

**Table S1.** Characteristics of the common garden experiments.

**Table S2.** Summary of the different allometric and linking functions tested for each species.

**Table S3.** Heat map for Pearson’s correlation coefficients, ρ, between *height* and *dbh*, together with the climatic and geographic variables from the experimental sites for the four species.

**Figure S1**. Principal Component Analysis (PCA) of pine populations (P) and growing sites (S).

**Figure S2.** The figure shows α_1p_ values for the four pine species studied, and their 95% credible intervals.

**Figure S3.** The figure shows α_2p_ values for the four pine species studied, and their 95% credible intervals.

**Figure S4.** The figure shows α_3p_ values for the four pine species studied, and their 95% credible intervals.

# Appendix text S1. Appropriateness of the use of estimated climate data instead of long-term average data originated in weather stations.

The Iberian Peninsula’s orography can change strongly in the scale of a few kilometers, and this can significantly influence climate in a specific local area. This fact makes the use of long-term average data from weather stations misleading to characterize sites which are far from those. For our case studies, we only had climate date from weather stations related to *Pinus halepensis* common gardens while, for the rest of the species’ common gardens, weather stations were too far (up to 60 km far).

Gonzalo-Jiménez's (2010) model is a robust climatic spatial interpolation. The author employed 1,350 thermo-pluviometry stations covering most of Spain, and included additional information to account for orography, slope, insolation, etc. He also employed a set of different calibration techniques to achieve a robust climatic model, which makes it a widely used climatic model.

In order to assess the suitability of the use of estimate climate data from Gonzalo-Jiménez's (2010) climate model, we present an example with the mentioned data –long-term data average– and a validation with data estimates used in the study (Table 1, below).

Table 1. Comparisons of climate values between long-term data average from weather stations and data estimates originated by Gonzalo-Jiménez's (2010) climate model (MAT_JG_).

| Weather  station name | Correspondence with the growing site | MAT (sd) | Nº years | MAT_JG_ |
| --- | --- | --- | --- | --- |
| Ademuz-Agro | Ademuz | 13.51 (0.64) | 9 | 13.7 |
| Sesga | Ademuz | 12.59 (0.55) | 11 | 13. 7 |
| Alcublas Polideportivo | Cucalón | 15.10 (0.00) | 1 | 14.5 |
| Fuente el Saz | Valdeolmos | 14.35 (0.37) | 12 | 13.6 |

We have compared mean annual temperature, MAT, from real weather stations along the period year from 1998 to 2011 (13 years) with MAT_JG_ data estimates from Gonzalo-Jiménez's (2010) (Table 1, above). Our estimates are quite similar to those calculated with long-term data average from real weather stations, i.e. climate data estimates are good enough to work with them. Since we lacked real data for the rest of the growing sites, we used the estimated climate data for all growing sites to have a more standard method.

**Table S1.** Characteristics of the common garden experiments.

| **Grow. Site** | **Year** | **Pop** | **Rep** | **Plot size** | | **Spacing** | **Surface** | | | **MMT** | | **AP** | | **Altitude** | |
| --- | --- | --- | --- | --- | --- | --- | --- | --- | --- | --- | --- | --- | --- | --- | --- |
| *P. sylvestris* |  |  |  |  |  | | |  |  | |  | |  | |  |
| Aragüés | 1991 | 22 | 4 | 16 | 2.5 × 2.5 | | | 0.88 | -4.10 | | 1,448 | | 1,424 | |  |
| Baza | 1991 | 16 | 4 | 16 | 2.5 × 2.5 | | | 0.64 | -2.30 | | 616 | | 1,789 | |  |
| Curueño | 1990 | 16 | 4 | 16 | 2.5 × 2.5 | | | 0.64 | -2.80 | | 835 | | 1,128 | |  |
| Gúdar | 1991 | 10 | 4 | 16 | 2.5 × 2.5 | | | 2.00 | -4.40 | | 677 | | 1,745 | |  |
| Manzanal | 1991 | 18 | 4 | 16 | 2.5 × 2.5 | | | 0.72 | -2.70 | | 883 | | 1,309 | |  |
| Navafría | 1990 | 19 | 4 | 16 | 2.5 × 2.5 | | | 0.76 | -4.20 | | 954 | | 1,743 | |  |
| *P. nigra* |  |  |  |  |  | | |  |  | | 0 | |  | |  |
| Rucandio | 1996 | 11 | 7 | 8 | 2.5 × 2.0 | | | 0.31 | -0.60 | | 692 | | 764 | |  |
| Herrera de P. | 1996 | 9 | 7 | 8 | 2.5 × 2.0 | | | 0.25 | -1.50 | | 538 | | 831 | |  |
| Sancedo 1 | 1996 | 10 | 7 | 8 | 2.5 × 2.0 | | | 0.28 | 0.80 | | 860 | | 729 | |  |
| Sancedo 2 | 1996 | 16 | 10 | 4 | 2.5 × 2.0 | | | 0.32 | 0.80 | | 860 | | 729 | |  |
| Sancedo 3 | 1997 | 14 | 10 | 4 | 2.5 × 2.0 | | | 0.28 | 0.80 | | 860 | | 729 | |  |
| Trespaderne2 | 1997 | 13 | 9 | 4 | 2.5 × 2.0 | | | 0.23 | 0.40 | | 686 | | 579 | |  |
| Aguilar de C. | 1996 | 17 | 10 | 4 | 2.5 × 2.0 | | | 0.32 | -2.10 | | 705 | | 1,012 | |  |
| La Granja | 1996 | 19 | 12 | 4 | 4.0 × 1.5 | | | 0.55 | -0.60 | | 460 | | 903 | |  |
| *P. pinaster* |  |  |  |  |  | | |  |  | | 0 | |  | |  |
| Acebo | 1966 | 52 | 4 | 16 | 2.5 × 2.5 | | | 2.08 | 2.20 | | 1220 | | 461 | |  |
| Cabañeros | 1967 | 52 | 4 | 16 | 2.5 × 2.5 | | | 2.08 | 0.00 | | 787 | | 1,023 | |  |
| Peñas N. | 1967 | 52 | 4 | 16 | 2.5 × 2.5 | | | 1.96 | 0.20 | | 549 | | 711 | |  |
| Riofrío | 1967 | 52 | 4 | 16 | 2.5 × 2.5 | | | 2.08 | 0.70 | | 719 | | 779 | |  |
| *P. halepensis* |  |  |  |  |  | | |  |  | |  | |  | |  |
| Ademuz | 1998 | 56 | 4 | 4 | 2.5 × 2.5 | | | 1.00 | -1.10 | | 367 | | 1,000 | |  |
| Altura | 1998 | 56 | 4 | 4 | 2.5 × 2.5 | | | 0.80 | 3.00 | | 517 | | 598 | |  |
| Valdeolmos | 1998 | 56 | 4 | 4 | 3.0 × 2.5 | | | 0.80 | 0.00 | | 475 | | 736 | |  |

**Grow. site:** Growing site corresponding to the common garden location. **Year**: Year of plantation. **Pop**: Number of populations tested. **Rep**: Number of replicates. **Plot Size**: Number of trees in the plot (or experimental unit). **Spacing**: Spacing of trees in the rows and columns (m), **Surface**: Total area (ha) of the common garden site. **MMT:** Minimum average temperature of the coldest month (ºC). **AP:** Annual precipitation (mm). **Altitude:** in (m)

**Table S2.** Summary of the different allometric and linking functions tested for each species. DIC criterion and ∆DIC (DIC_i_ – DIC_min_) provides objective measures of the strength of the empirical support for the competing models. The model with the lowest DIC is highlighted in **bold** letters for each species.

| **Species** | **Allometric function** | **Linking function** | **DIC** | **Δ DIC** |
| --- | --- | --- | --- | --- |
| *P. sylvestris* | Linear | Normal | 52430 | 2630 |
|  | Power | Normal | 53270 | 3470 |
|  | Gompertz | Normal | 60590 | 10790 |
|  | **Linear** | **Log Normal** | **49800** | **0** |
|  | Power | Log Normal | 52360 | 2560 |
|  | Gompertz | Log Normal | 58940 | 9140 |
| *P. nigra* | Linear | Normal | 27540 | 1180 |
|  | Power | Normal | 28190 | 1830 |
|  | Gompertz | Log Normal | 26590 | 230 |
|  | Linear | Log Normal | 30950 | 4590 |
|  | **Power** | **Log Normal** | **26360** | **0** |
|  | Gompertz | Normal | 35460 | 9100 |
| *P. pinaster* | Linear | Normal | 119800 | 300 |
|  | Power | Normal | 119700 | 200 |
|  | Gompertz | Normal | 124200 | 4700 |
|  | Linear | Log Normal | 119700 | 200 |
|  | **Power** | **Log Normal** | **119500** | **0** |
|  | Gompertz | Log Normal | 130700 | 11200 |
| *P. halepensis* | Lineal | Normal | 15350 | 230 |
|  | Power | Normal | 15430 | 310 |
|  | Gompertz | Normal | 19050 | 3930 |
|  | Linear | Log Normal | 15240 | 120 |
|  | Power | Log Normal | 15230 | 110 |
|  | **Gompertz** | **Log Normal** | **15120** | **0** |

**Table S3.** Heat map for Pearson’s correlation coefficients, ρ, between *height* and *dbh*, together with the geographic and climatic variables from the growing sites for the four species (dark grey color indicates high positive correlation coefficients, light grey indicates high negative, and white indicates low). The selected variables for the final model are shown in *italics* and *underlined* letters. **Bold** numbers means significant correlations at p < 0.05.

|  | *P. sylvestris* | | *P. nigra* | | *P. pinaster* | | *P. halepensis* | |
| --- | --- | --- | --- | --- | --- | --- | --- | --- |
|  | *dbh* | *height* | *dbh* | *height* | *dbh* | *height* | *dbh* | *height* |
| Longitude | **0.58** | **0.57** | **-0.65** | **-0.62** | **-0.28** | **-0.35** | **0.39** | **0.46** |
| Latitude | **0.12** | **0.19** | **0.15** | **0.07** | **0.21** | **0.25** | **-0.40** | **-0.48** |
| Altitude | **0.25** | **0.18** | **-0.35** | **-0.32** | **-0.37** | **-0.47** | **-0.69** | **-0.73** |
| MMTJan | **-0.61** | **-0.62** | **0.45** | **0.45** | **0.32** | **0.40** | **0.73** | **0.80** |
| MMTFeb | **-0.63** | **-0.66** | **0.48** | **0.48** | **0.24** | **0.31** | **0.73** | **0.80** |
| MMTMar | **-0.67** | **-0.69** | **0.53** | **0.53** | **0.19** | **0.25** | **0.73** | **0.80** |
| MMTApr | **-0.70** | **-0.71** | **0.40** | **0.41** | **0.33** | **0.42** | **0.73** | **0.80** |
| MMTMay | **-0.39** | **-0.42** | **0.46** | **0.48** | **0.21** | **0.28** | **0.58** | **0.66** |
| MMTJun | **-0.04** | **-0.06** | **0.48** | **0.50** | **0.10** | **0.15** | **0.48** | **0.56** |
| MMTJul | **-0.05** | **-0.11** | **0.56** | **0.58** | **-0.12** | **-0.12** | **0.69** | **0.76** |
| MMTAug | **-0.13** | **-0.20** | **0.53** | **0.56** | **-0.06** | **-0.05** | **0.57** | **0.65** |
| MMTSep | **-0.54** | **-0.60** | **0.57** | **0.58** | **-0.03** | -0.01 | **0.66** | **0.74** |
| MMTOct | **-0.68** | **-0.70** | **0.56** | **0.55** | **0.10** | **0.14** | **0.72** | **0.79** |
| MMTNov | **-0.68** | **-0.70** | **0.53** | **0.53** | **0.12** | **0.17** | **0.72** | **0.79** |
| MMTDec | **-0.61** | **-0.62** | **0.40** | **0.39** | **0.31** | **0.39** | **0.73** | **0.80** |
| MWTJan | **-0.31** | **-0.33** | **0.32** | **0.37** | **0.40** | **0.51** | **0.55** | **0.63** |
| MWTFeb | **-0.50** | **-0.51** | **0.33** | **0.37** | **0.36** | **0.46** | **0.61** | **0.69** |
| MWTMar | **-0.64** | **-0.64** | **0.33** | **0.37** | **0.29** | **0.38** | **0.22** | **0.29** |
| MWTApr | **-0.68** | **-0.68** | **0.17** | **0.22** | **0.35** | **0.45** | **-0.51** | **-0.58** |
| MWTMay | **-0.62** | **-0.63** | 0.01 | **0.07** | **0.32** | **0.40** | **-0.67** | **-0.75** |
| MWTJun | **-0.49** | **-0.52** | **0.12** | **0.18** | **0.16** | **0.22** | **-0.72** | **-0.79** |
| MWTJul | **-0.49** | **-0.54** | **0.07** | **0.14** | 0.05 | **0.08** | **-0.69** | **-0.76** |
| MWTAug | **-0.39** | **-0.44** | -0.05 | 0.02 | **0.06** | **0.10** | **-0.73** | **-0.79** |
| MWTSep | **-0.45** | **-0.49** | **-0.08** | -0.01 | **0.13** | **0.18** | **-0.73** | **-0.80** |
| MWTOct | **-0.46** | **-0.48** | **0.11** | **0.17** | **0.27** | **0.35** | **-0.25** | **-0.23** |
| MWTNov | **-0.27** | **-0.28** | **0.27** | **0.32** | **0.37** | **0.47** | **0.59** | **0.67** |
| MWTDec | **-0.26** | **-0.29** | **0.22** | **0.26** | **0.47** | **0.59** | **0.67** | **0.75** |
| MTJan | **-0.47** | **-0.49** | **0.42** | **0.44** | **0.41** | **0.52** | **0.69** | **0.77** |
| MTFeb | **-0.61** | **-0.63** | **0.42** | **0.44** | **0.34** | **0.44** | **0.72** | **0.79** |
| MTMar | **-0.69** | **-0.69** | **0.47** | **0.49** | **0.26** | **0.34** | **0.73** | **0.80** |
| MTApr | **-0.69** | **-0.69** | **0.26** | **0.31** | **0.35** | **0.44** | **0.67** | **0.71** |
| MTMay | **-0.60** | **-0.62** | **0.21** | **0.27** | **0.30** | **0.39** | **-0.56** | **-0.57** |
| MTJun | **-0.35** | **-0.38** | **0.31** | **0.36** | **0.15** | **0.20** | **-0.72** | **-0.78** |
| MTJul | **-0.34** | **-0.39** | **0.33** | **0.38** | **-0.01** | 0.01 | **-0.68** | **-0.76** |
| MTAug | **-0.30** | **-0.36** | **0.22** | **0.28** | **0.00** | **0.02** | **-0.71** | **-0.77** |
| MTSep | **-0.50** | **-0.55** | **0.23** | **0.29** | **0.07** | **0.11** | **-0.60** | **-0.62** |
| MTOct | **-0.57** | **-0.59** | **0.39** | **0.43** | **0.21** | **0.28** | **0.66** | **0.74** |
| MTNov | **-0.51** | **-0.53** | **0.45** | **0.47** | **0.28** | **0.36** | **0.69** | **0.77** |
| MTDic | **-0.43** | **-0.45** | **0.32** | **0.34** | **0.45** | **0.56** | **0.72** | **0.79** |
| *AP* | **0.65** | **0.70** | **0.42** | **0.37** | **0.28** | **0.34** | **0.67** | **0.71** |
| WintP | **0.53** | **0.59** | **0.52** | **0.47** | **0.29** | **0.36** | **0.27** | **0.25** |
| SpringP | **0.77** | **0.79** | **0.24** | **0.19** | **0.35** | **0.43** | **0.58** | **0.60** |
| SumP | **0.62** | **0.66** | **-0.46** | **-0.49** | 0.02 | **0.00** | **-0.05** | -0.01 |
| AutP | **0.59** | **0.65** | **0.44** | **0.39** | **0.31** | **0.38** | **0.73** | **0.78** |
| MAT | **-0.57** | **-0.60** | **0.47** | **0.50** | **0.13** | **0.19** | **0.68** | **0.76** |
| WT | **-0.24** | **-0.30** | **0.36** | **0.41** | **-0.10** | **-0.11** | **-0.71** | **-0.79** |
| MWT | **-0.42** | **-0.47** | **0.13** | **0.19** | **-0.06** | **-0.05** | **-0.70** | **-0.77** |
| MT | **-0.47** | **-0.49** | **0.49** | **0.50** | **0.37** | **0.47** | **0.70** | **0.77** |
| *MMT* | **-0.61** | **-0.62** | **0.50** | **0.50** | **0.32** | **0.40** | **0.73** | **0.80** |

**MMT**# is the mean minimum temperature of the month #; **MWT**# is the mean maximum temperature of the month #; **MT**# is the mean temperature of the month #; **WintP** is total winter precipitation; **SpringP** is total spring precipitation; **SumP** is the total summer precipitation; **AutP** is total autumn precipitation; **AP** is the annual precipitation; **MAT** is the mean annual temperature; **WT** is the mean temperature of the warmest month; **MWT** is the mean maximum temperature of the warmest month; **MT** is the mean temperature of the coldest month; and **MMT** is minimum average temperature of the coldest month.

**Figure S1**. Principal Component Analysis (PCA) of pine populations (P) and growing sites (S) based on their climatic variables (see Material and Methods) of the following four pine species: *Pinus halepensis* (Ph), *P. nigra* (Pn), *P. pinaster* (Pp) and *P. sylvestris* (Ps).

|  |
| --- |
|  |
|  |
|  |

**Figure S2.** The Fig. shows α_1p_ values (represented by diamonds) for the four pine species studied, and their 95% credible intervals (represented by triangles). X-axis indicates the population name. The hyperparameter, µ_1,_ is represented with a black diamond.

**Figure S3.** The Fig. shows α_2p_ values (represented by diamonds) for the four pine species studied, and their 95% credible intervals (represented by triangles). X-axis indicates the population name. The hyperparameter, µ_2,_ is represented with a black diamond.

|  |
| --- |
|  |
|  |
|  |

**Figure S4.** The Fig. shows α_3p_ values (represented by diamonds) for the four pine species studied, and their 95% credible intervals (represented by triangles). X-axis indicates the population name. The hyperparameter, µ_3,_ is represented with a black diamond.
